# Bread Wheat in Space Flight: Is There a Difference in Kernel Quality?

**DOI:** 10.3390/plants13010073

**Published:** 2023-12-25

**Authors:** Tatiana S. Aniskina, Kirill A. Sudarikov, Margarita A. Levinskikh, Alexander A. Gulevich, Ekaterina N. Baranova

**Affiliations:** 1 N.V. Tsitsin Main Botanical Garden of Russian Academy of Sciences, 127276 Moscow, Russia; 2 Russian State Agrarian University—Moscow K.A. Timiryazev Agricultural Academy (RSAU-MTAA), Timiryazevskaya 49, 127434 Moscow, Russia; sudarikov@zolshar.ru; 3 Institute of Development Strategy, 101000 Moscow, Russia; 4 Institute of Biomedical Problems, 123007 Moscow, Russia; r.levinskikh@gmail.com; 5 All-Russia Research Institute of Agricultural Biotechnology, Timiryzevskaya 42, 127550 Moscow, Russia; a_gulevich@mail.ru

**Keywords:** *Triticum aestivum* L., wheat kernel, starch granules, growing in space conditions, space station, hyperspectral imaging

## Abstract

Planning long-term space flights necessarily includes issues of providing food for the crew. One of the areas of research is the development of technologies for independent production of food by the crew. Extensive research on lettuce has confirmed that the “space production” of lettuce is not inferior to that on Earth, even in the absence of gravity, but the same deep understanding of the quality of grain crops has not yet been achieved. Therefore, the goal of our work is to establish whether the conditions for growing wheat in outer space without gravity affect the weight and basic parameters of the grain, and whether this leads to increased asymmetry of the kernel and distortion of the starch composition. The objects of the study were wheat (*Triticum aestivum* L.) kernels of the Super Dwarf cultivar. Of which, 100 kernels matured in outer space conditions in the Lada growth chamber on the International Space Station (ISS), and 85 kernels of the control wheat grown in a similar growth chamber under terrestrial conditions. It has been established that kernels from ISS have significant differences to a smaller extent in weight, area, length, and width of the kernel. However, the kernels under both conditions were predominantly large (the average weight of a kernel in space is 0.0362 g, and in terrestrial conditions—0.0376 g). The hypothesis that the level of fluctuating asymmetry will increase in outer space was not confirmed; significant differences between the options were not proven. In general, the kernels are fairly even (coefficients of variation for the main parameters of the kernel are within 6–12%) and with a low or very low level of asymmetry. The length of starch granules of type A in filled and puny kernels is significantly greater in kernels from ISS than in the control, and in terms of the width of starch granules B and roundness indices, both experimental variants are the same. It can be assumed that the baking qualities of earthly kernels will be slightly higher, since the ratio of type B starch granules to type A is 5–8% higher than on the ISS. Also, the width of the aleurone layer cells in mature kernels was significantly inferior to the result obtained on Earth. The work proposes a new method for establishing the asymmetry of kernels without a traumatic effect (in early works, it was supposed to study asymmetry in transverse sections of the kernels). Perhaps this will make it possible to further develop a computer scanning program that will determine the level of asymmetry of the wheat fruit.

## 1. Introduction

When planning piloting beyond our planet and long-term space exploration, the possibility of obtaining food in a new place is taken into account, because delivery to Mars, the Moon, or a space station will be unreasonably high in cost, long, and irregular [1]. Therefore, ongoing work is underway to improve greenhouses with a controlled environment to ensure sustainable food security, as well as research into plants suitable for such conditions, primarily dwarf plants, and those with accelerated development [2]. Plants are selected that are resistant to high levels of ethylene, with a short period of obtaining marketable products, compact size, high productivity, and self-reproduction (from seed to seed) [3]. Plants in space can serve not only as a source of food, but also for the in situ production of pharmaceuticals, air regeneration, water recycling, and improving the mental health of astronauts [4,5,6].

In near space conditions, technologies have already been developed for producing plants from seed to seed using model plants, for example, *Arabidopsis thaliana* [7] and *Brassica rapa* [8], as well as cultivated annual plants of various families.

The main crops consumed by humans are wheat, rice, corn, and sorghum [9]. Previously, attempts to grow cereals were sporadic and often ended in failure [2,10]. Currently, methods for cultivating wheat [11,12] have been developed (sorghum, due to the absence of dwarf forms, remains not yet relevant). The breakthrough in the cultivation of these crops is associated with overcoming pollination problems associated with certain development issues and gas requirements, particularly ethylene [13].

Currently, the range of dwarf forms of rice and wheat makes it possible to achieve sustainable results, both on the ISS [10] and on the name of the Tiangong Chinese station [14].

Kernel parameters such as length, width, weight, and shape make the most significant contribution in yield assessment [15]. The composition and ratio of different types of starch grains in the endosperm, as well as the protein content, mainly related to the quantity and characteristics of the formation of the aleurone layer, determine the quality criteria of the resulting flour and other processed products [2]. Quality assessment based on completion and the symmetry of kernels makes flour production predictable.

Despite the fact that the very issue of obtaining a crop of cereals grown under near space conditions has now already been resolved, even with the use of genetic analysis [16,17], the issue of assessing crop quality, and, in particular, the symmetry of the grains still remains unstudied.

The quality of the harvest is already checked on Earth. To identify clear patterns and long-term consequences, it is necessary to create express methods for diagnosing seed quality already in orbit. This must be accompanied by the development of non-destructive assessment methods to maintain the feasibility of using seeds in experiments. One such method could be phenotypic assessment and assessment of shape and color based on various pre-assessed indices. There have not yet been any studies on the asymmetry of plant organs grown in space, and we could not find the results of using hyperspectral analysis for these purposes.

Due to the characteristics of cultivation and the possible action of various factors, wheat, grown in a growth chamber with conditionally full compliance with temperature, lighting, and humidity on the ISS, will be subject to a number of stressful influences to which they are poorly adapted: 24 h light; a constant, relatively warm temperature; and poor light quality. The major difference between the two environments is gravity. Because the wheat plants have phototropism, the effects on gravitropism on plant development were studied. Kernels of smaller weight, length, width, area, and perimeter will be formed in a such condition. This assumption was designated as hypothesis A.

It was also hypothesized that the level of wheat kernel asymmetry would be higher on the ISS board (hypothesis B). A visual method for assessing the asymmetry of the kernel on a cross section of a kernel was previously proposed [18]. However, since the amount of grain collected on the ISS was limited, it was also necessary to develop a method for asymmetry evaluation without cutting into these unique accessions. A hypothesis was also assumed (hypothesis C) that there is a relationship between the asymmetry of the kernel and its main parameters, and the stronger the asymmetry, the lower the weight, since asymmetry is probably characteristic of puny (unfulfilled, immature) kernels.

## 2. Results

The wheat plants obtained during the full-size experiment on the ISS and in the ground laboratory Lada greenhouse were harvested after reaching the full maturity stage. The kernels were husked from the ears, weighed, and stored under the same conditions. To determine the natural range of differences, visually determined parameters were assessed. At the same time, it was found that some of the kernels were larger in size and had a fully formed shape; some had signs of stunting, namely an uneven and asymmetrical shape. This scatter is typical for kernels formed on spikelets in different zones of the ear, wherein well-filled kernels were formed in the spikelets of the central part of the ear, and puny kernels were formed in the upper and lower parts.

Another morphological phenomenon that needed to be taken into account was the presence of a through hole in some of the kernels, probably associated with unknown variability and peculiarities of the formation of the middle part of the endosperm. This feature can be noted for part of the grains as a characteristic feature both for kernels of Super Dwarf wheat grown in the field, and for kernels of wheat grown in Lada greenhouses under the conditions of a ground experiment and in low-Earth orbit under the conditions of the ISS. This hole was observed in both well-filled and puny kernels, that is, these signs were not related. The identified natural fluctuations of the signs of “puny/well-fulfilled” and the “presence/absence of a through hole” were taken into account when analyzing kernels and forming a sample for analysis.

### 2.1. Descriptive Statistics of Kernels

It was found that wheat grown on the ISS has significantly smaller kernels by weight (by 0.0014 g), area (by 1.68 mm^2^), length (by 0.45 mm), and width (by 0.11 mm). However, the differences between the perimeter, depth, and roundness of the kernels are not statistically significant (Table 1), which partially confirms hypothesis A.

**Figure 1:**
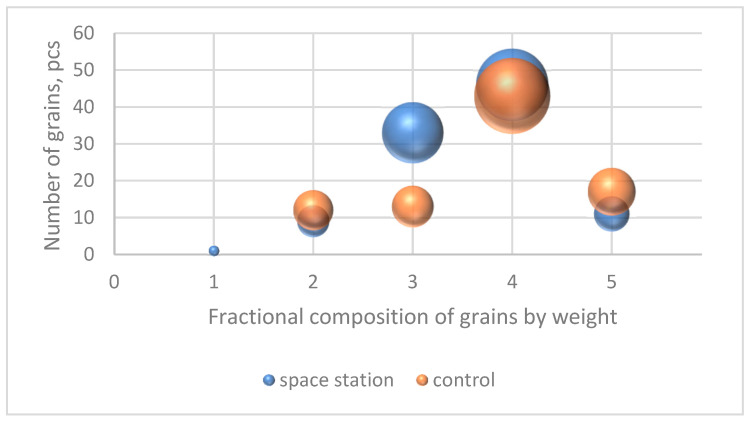
Distribution of frequencies of occurrence (in pieces) of accessions in samples with the ISS and in the control variant by weight, where the height of the bubble corresponds to the number of kernels in the sample, and the diameter of the bubble corresponds to the percentage of kernels in the sample. The horizontal axis indicates the fractional composition: 1—very low weight (0.0208–0.0259 g), 2—small (0.0260–0.0311 g), 3—medium (0.0312–0.0363 g), 4—large (0.0364–0.0415 g), 5—very large (0.0416–0.0467 g).

Based on kernel weights, large ones predominate in both samples (0.0364–0.0415 g), in the sample with the ISS there are 46 kernels out of 100 and in the control 43 out of 85 accessions. There are 33 medium-sized (0.0312–0.0363 g) kernels with ISS and 13 control ones (Figure 1). Very large (0.0416–0.0467 g)—11 accessions from the ISS and 17 in the control variant.

**Figure 2:**
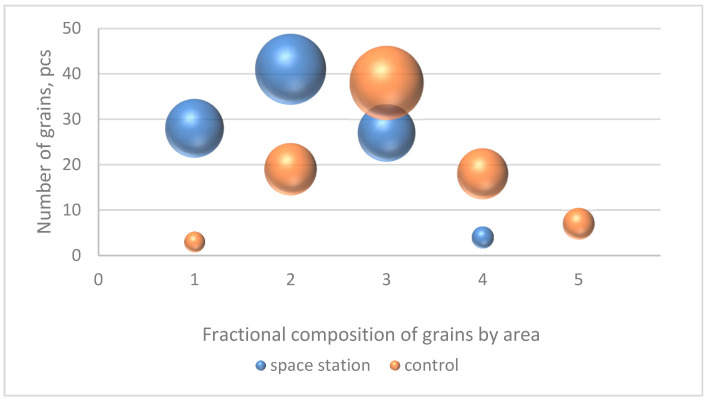
Distribution of frequencies of occurrence (in pieces) of kernels in samples from the MCS and in the control variant by area, where the height of the bubble location corresponds to the number of pieces of kernels in the sample, and the diameter of the bubble corresponds to the percentage of the size of kernels in the sample. The horizontal axis indicates the fractional composition: 1—very small area (9.2–10.8 mm^2^), 2—small (10.8–12.4 mm^2^), 3—medium (12.4–14.0 mm^2^), 4—large (14.0–15.6 mm^2^), 5—very large (15.6–17.2 mm^2^).

An analysis of the frequency of occurrence of kernels by area (Figure 2) showed that the kernels harvested on the ISS are mostly small in size (10.8–12.4 mm^2^), very small (9.2–10.8 mm^2^), and medium (12.4–14.0 mm^2^)—41, 28, and 27 pieces out of 100, respectively. In the control variant, kernels of medium, small, and large (14.0–15.6 mm^2^) sizes predominate—38, 19, and 18 pieces out of 85, respectively.

### 2.2. Checking for Kernel Asymmetry Using Non-Traumatic Methods

A study of the first non-traumatic method for determining the asymmetry of kernel using the methods of perpendiculars to the axis of symmetry at points of 50, 25, and 75% of the kernel length on a sample of 15 kernels did not establish significant differences between the left and right sides of the kernel. Increasing the sample to 30 kernels led to an increase in the significance of the Mann–Whitney and Kruskal–Wallis coefficients, indicating that there were no significant differences between the left and right sides of the kernel. That is, this method is not able to detect asymmetry even in those grains in which it is visually present.

The second method of comparing the length of the left and right sides of the kernel for all samples of the Super Dwarf cultivar using the *t*-test method showed that there are no differences only between the distances from the tops of the folds of the left and right sides of the endosperm to the axis of symmetry. When examining the samples separately, it was found that the left and right sides differ along all perpendiculars in kernels with ISS, while in the control variant there are no differences only between the distances from the tops of the folds of the left and right sides of the endosperm to the axis of symmetry.

### 2.3. Comparison of Asymmetry Indices Obtained from a Section of a Kernel and a Non-Traumatic Method

**Figure 3:**
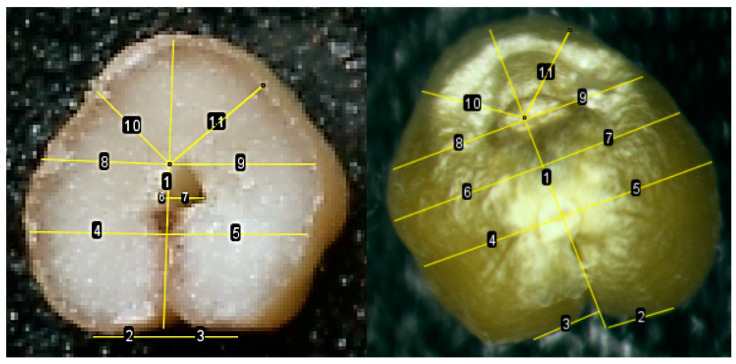
Comparison of the lengths of perpendiculars on a section of a kernel and a non-traumatic method. Thick lines indicate perpendiculars without significant differences.

Comparison of the lengths of perpendiculars to the axis of symmetry showed that there are no significant differences between the length of L1 on the cut and L1 in a non-traumatic way; the lengths of L1’, L2, L2’, L5, L5’ are also statistically the same when tested using both the Kolmogorov–Smirnov method and Mann–Whitney test (Figure 3). The lateral lobes form morphologically slightly before the central lobe. Thus, kernel defects and environmental stress often affect the central lobe (the angle between lines 10 and 11) more than the lateral lobes.

### 2.4. Relationship between the Main Kernel Parameters and Asymmetry Indicators Obtained in a Non-Traumatic Manner

In terms of the direction of asymmetry, in 42 kernels from ISS, a pronounced shift of indices to the left was observed. This may indicate that these kernels were located on the right in the ear. Also, in nine kernels, there was a clear shift to the right, thirty-two kernels were symmetrical, and, in seventeen kernels, this indicator could not be reliably determined (for example, two indices were shifted to the left, two indices were shifted to the right, and one was symmetrical). The control variant had 38 kernels with indices shifted to the left, 9—to the right, 27 have pronounced symmetry, and in 11 kernels the indicator could not be unambiguously determined.

**Figure 4:**
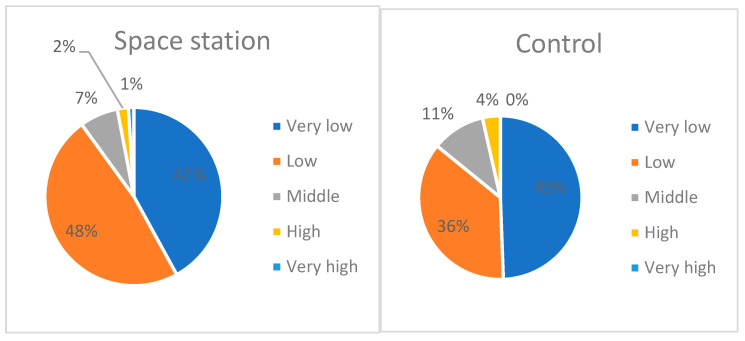
Correlation of kernels according to the level of fluctuating asymmetry in two experimental variants (ground control and space station).

According to the fluctuating asymmetry (FA) scale developed by Aniskina et al. (2023) [18], kernels from the ISS and control ones have a low and very low level of asymmetry (Figure 4). For example, 48 kernels with a low level of asymmetry and 42 ones with a very low level were received from the ISS, while in the control ground variant there were 31 and 42 of these, respectively. The average level of FA (index 0.132–0.192) was in seven kernels from the ISS and in nine from the control ground variant, a high level of FA was in two kernels from the ISS and in three kernels from the ground experiment, and a very high (0.254–0.314) FA level was observed only in one kernel from the ISS. The *t*-test did not establish significant differences in fluctuating asymmetry between kernels from ISS and the control variant; that is, hypothesis B was not confirmed.

Correlation analysis of traits showed that there was a significant strong relationship between FA and relative asymmetry index 4 in both the control and cosmic kernels. The direction of asymmetry was strongly associated with index 3 in both treatments, and with index 5 in kernels from ISS. That is, to minimize labor costs and speed up calculations, it is enough to take measurements from the edges to the axis through the radicle (L4 and L4’) and from the edges to the axis through the hole (L3 and L3’); in addition, you can measure the distance from the edge to the axis at an angle of 45% (L5 and L5’).

The relationship between the weight of the kernels, area, perimeter, roundness, length, and width with asymmetry indicators has not been established. That is, hypothesis C turned out to be incorrect.

### 2.5. Comparison of Starch Granules in Kernels from the ISS and from Ground Experiment

It is customary to divide starch granules into type A (size < 10 µm) and type B (>10 µm). A comparison of the coefficients of variation for a scattering of starch granules of type A in kernels from the ISS and from Earth (ground experiment) showed that the greatest variability in length and width was present in matured (filled) kernels (Table 2). Granules of type B are more aligned, for example, the variability in the width of puny kernels was 46% for granules of type A, and half as much for granules of type B (21%). The indices of the length and width of type A granules for kernels from the ISS and from Earth are the same—11 and 14% for mature kernels, and 12 and 11% for puny ones.

The length of type A starch granules in matured (filled) kernels from the ISS is significantly longer than in kernels from the ground experiment (Table 3). Also, the length and width of type B granules in puny kernels from Earth were significantly smaller. The roundness indices are the same within all types of granules, as well as the width of type A granules.

**Figure 5:**
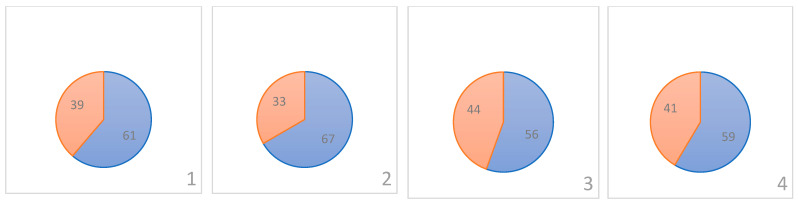
Distribution of starch grains by type of starch granules in %. The blue area is type A starch granules, the orange area is type B starch granules. Diagram 1—ISS, matured kernels; 2—ISS, puny kernels; 3—Earth, matured kernels; 4—Earth, puny kernels.

The ratio of starch granules type A and B affects the baking properties of flour. An analysis of the ratio of granules showed that in kernels from the ISS there is 5% less type B starch in mature kernels than in the control ground variant, as well as in puny kernels—the difference with the Earth is 8% (Figure 5). In general, the distribution of the two types of granules in kernels from the Earth treatment was close to an equal ratio, but in kernels from the ISS there was a significant bias towards type A granules.

Cells of the aleurone layer in wheat kernels from the ground treatment and from the ISS did not have significant differences in length (Table 4). However, the width of the cells in mature kernels from the ISS was significantly smaller (Table 4).

When analyzing the qualitative characteristics of starch granules, median sections differing in shape, aspect ratio, and the presence of extensions in the central part of the kernel were used. The ratio of sizes, number of starch granules of various types, and their location are associated with varietal characteristics, predicted flour quality, cultivation characteristics, and the presence of pests. Due to the fact that under space flight conditions, as in laboratory conditions, the main control indicators such as humidity, lighting, nutrition were the same, and pests and diseases were not identified, it can be assumed that the differences could be caused by the specific location of the greenhouse. The shape, the ratio of the number of starch granules, as well as the binder and matrix, which is the remains of the cytoplasm, are indicators of the quality of the resulting flour and starch.

The starch granules in the endosperm of the kernels had a structural organization characteristic of wheat. Large starch granules were inclusions formed in plastids and they formed a flat spindle-shaped structure, which was expressed in depressions located along the widest part. Smaller grains are also formed in plastids and their quantity affects the quality of starch and, therefore, flour. The forming granules in fully completed kernels under terrestrial conditions had a smooth surface with a depression on the side and smaller starch inclusions of type B. In kernels that have impaired development and pronounced asymmetry of the two sides, apparently associated with the peculiarities of formation, lack of nutrients, local disruption of the supply of nutrients, some disturbances in the shape, and ratio of starch granules deposits could be observed.

**Figure 6:**
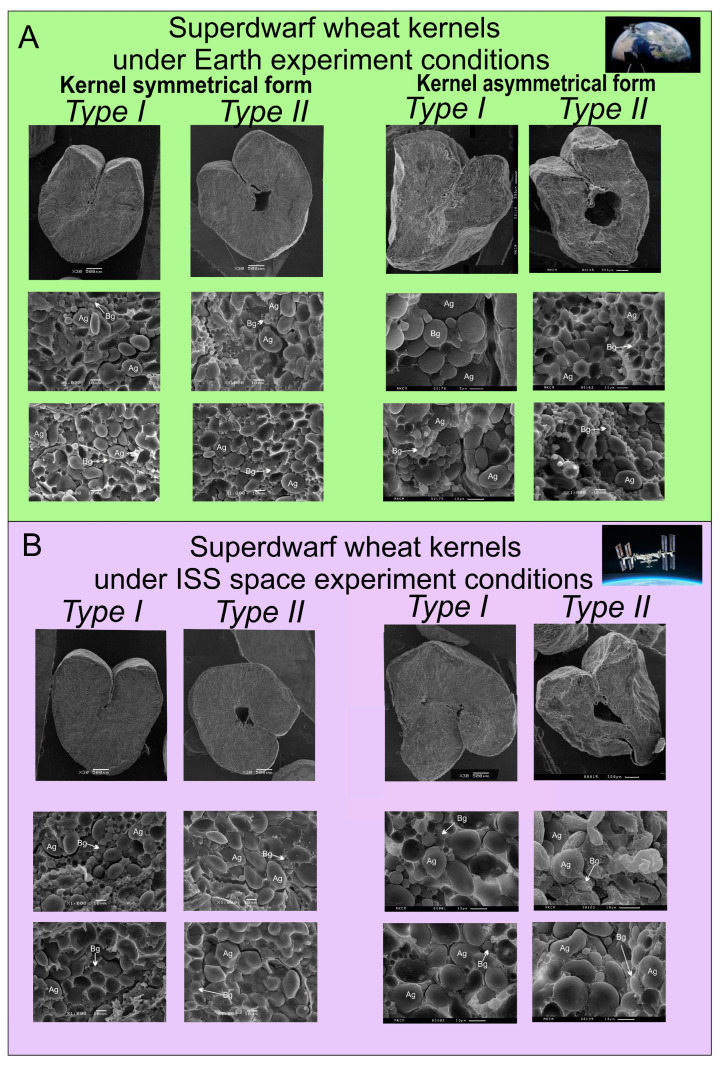
Scanning transmission electron microscopy of cross sections of air-dried Super Dwarf wheat kernels grown in the Lada growth chamber under space flight conditions on Earht (**A**) and under laboratory experiment conditions on the ISS (**B**). Shown are images of sections and enlarged fragments of the central part of the endosperm of kernels that had uniform development and a symmetrical shape (left), as well as kernels that had pronounced asymmetry (right). Additionally, two types of cores are given that are characteristic of this variety—having a dense structure in the middle part and characterized by an expanded through the hole. Enlarged fragments show typical areas of endosperm cells with two types of starch granules characteristic of wheat (A-type (Ag) and B-type (Bg)).

Under cultivation conditions in ISS, small changes in shape in the granules, and significant disturbances in the quantity, ratio of granule types, and sizes in granules with an uneven and incompletely formed system of deposition of reserve substances was observed (Figure 6). A breakdown of the binding components could also be observed.

### 2.6. Comparison of Averaged Spectral Profiles Obtained Using Hyperspectral Imaging

**Figure 7:**
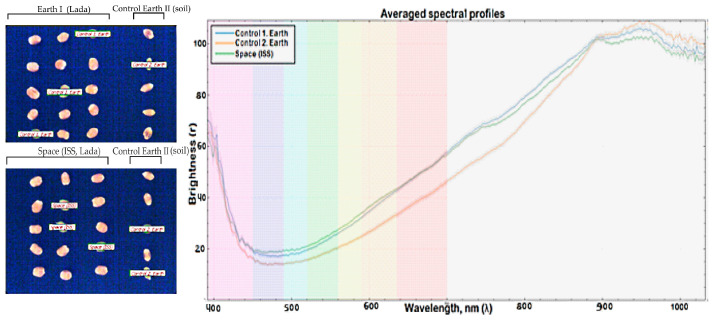
Panel with fixed kernels used for hyperspectral analysis and the result of averaged data. The kernels of fresh harvest and kernels obtained in Lada greenhouses in laboratory conditions and on the ISS are presented.

A series of photographs were taken, in each of which three kernels from each group were randomly selected. Next, graphs were obtained by averaging the spectral profiles (Figure 7). Visually, there were no obvious differences between these groups in the RGB format (R-640 nm, G-550 nm, B-460 nm).

Based on the obtained averaged spectral profiles, we can conclude that the accessions from the control ground treatment and the treatment from ISS are as similar as possible to each other and their graphs almost completely repeat each other. The kernels in the ground treatment (Control 2. Earth) differed from the first two groups of kernels in that they had weaker reflection (r) in the red and near-infrared spectrum (650–800 nm).

### 2.7. Comparison of NDVI Indices of Kernels Obtained Using Hyperspectral Imaging

**Figure 8:**
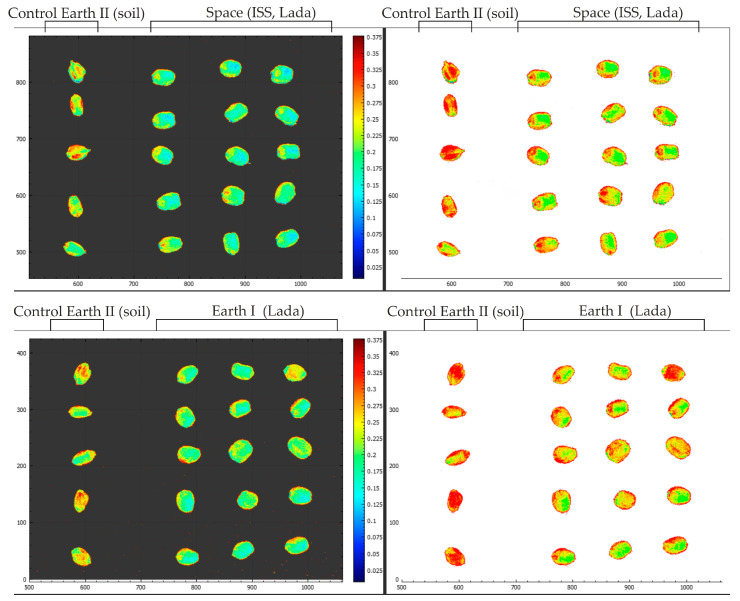
Results of hyperspectral analysis using the NDVI index to visualize kernels. Fresh harvest kernels and kernels obtained in Lada greenhouses in laboratory conditions and on the ISS are presented.

The NDVI index was taken in the range from 0 to 3.75 for maximum clarity. In all the images below with the NDVI index overlaid, the kernels are from the Control 2. Earth treatment are visible on the left side (Figure 8). Based on these images, we can conclude that kernels after long-term storage have a lower vegetation index in contrast to kernels obtained from new resowing. The NDVI index was taken in the range from 0 to 3.75 for maximum visibility. 

### 2.8. Comparison of Approximation Indices of the Kernel Spectral Profile to a Given Anthocyanin Standard

**Figure 9:**
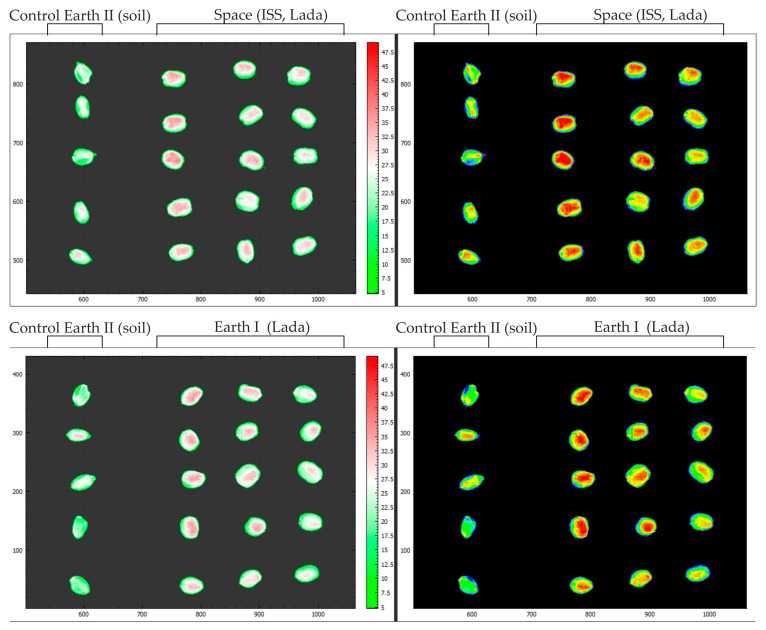
Results of hyperspectral analysis using the method of interpolating the spectral profile onto a reference anthocyanin profile for visualizing kernels. The kernels of fresh harvest and kernels obtained in Lada growth chamber in laboratory conditions and on the ISS are presented.

**Figure 10:**
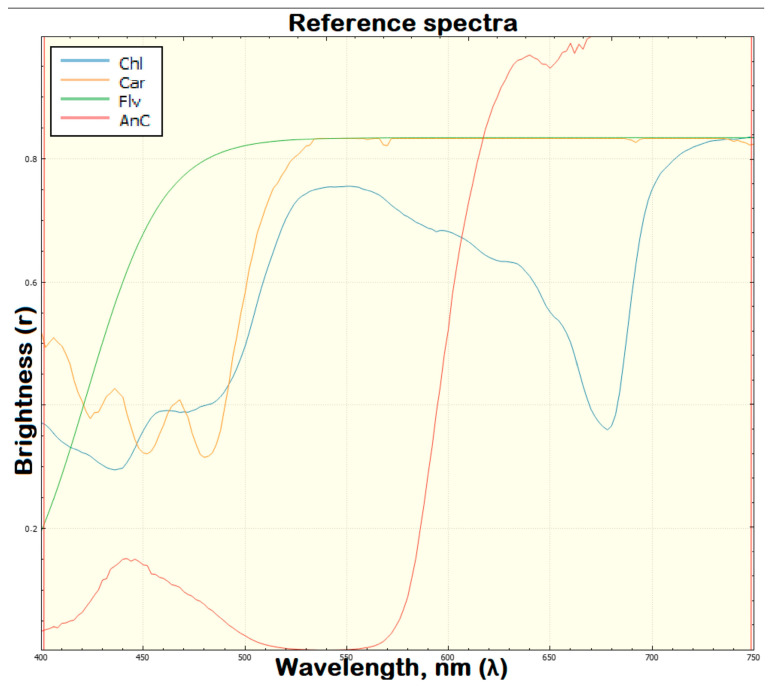
Reference brightness spectra from 0 to 1 used to superimpose the scanned brightness by spectral profile interpolation onto the reference anthocyanin profile.

The reference spectra are reduced to a brightness level from 0 to 1, therefore, to superimpose the brightness we obtained, the method of interpolating the spectral profile onto the reference anthocyanin profile was used (Figure 9). To obtain a single profile approximation index, an index was obtained, which was subsequently superimposed on the images to show the anthocyanin ratios in the original hyperspectral image. In all the images below with the Anthocyanin Index overlaid, AnC can be seen on the left side of the Control 2, Earth panel (Figure 9). From the analysis of the images with the index superimposed on them, it follows that the anthocyanin content in the kernels increases during storage (Figure 10).

## 3. Discussion

Providing for people during long space expeditions, when working on remote objects of deep and near space, poses significant challenges for agronomic support and necessarily encourage the creation of water reclamation systems, efficient use of nutrients, and a closed cycle of plant cultivation with mandatory reproduction of plant crops from seed to seed [6,19].

For the first time, this study describes a comparison of Super Dwarf wheat grown in a growth chamber in near space conditions on the ISS with wheat grown in a growth chamber on Earth. The Lada chamber was carefully designed so that the main difference between the treatments was gravity. The ISS-grown plants appeared visually normal, likely because phototropism responses under both treatments were similar. Since no major morphological changes in plant growth were observed, the accumulative effects of gravity were evaluated in the kernels produced under the two treatment conditions. Because the experiment could not be replicated, detailed studies were conducted to determine if differences between kernels produced in the two environments exist. However, the nature of those studies was restricted by both small sizes and non-damaging evaluations. It has been found that the simple scanning of a scattering of kernels is not suitable for a non-traumatic method of asymmetry determination. The presence of asymmetry can be detected by measuring the length of the left and right parts of the kernel, scanning (or photographing under a microscope) the base of the kernel. That is, of the two proposed methods, only the second is suitable—measuring the lengths of perpendiculars to the axis of symmetry through the point of attachment of the kernel and the zone of closure of the two lobes of the kernel. It was found that the weight, area, length, and width of the kernels from the plants grown in the ISS are less than those in the ground control, with statistically identical perimeters and roundness. Hypothesis A, that all parameters would be significantly lower, was put forward, but the hypothesis was only partially confirmed. A similar phenomenon was observed in a study of Arabidopsis seed coats—the size and number of cells inside the seed coat were statistically the same [20], as well as the number of lettuce leaves [14]. Also, the hypothesis regarding the distortion of the shape of the kernel (Hypotheses B and C) were not confirmed, although early studies mentioned various types of deviations, for example, chromosome breakage [21], inability to produce seeds [22,23], distorted and non-viable embryos [24], and morphological abnormalities [25]. This might be due to improved systems for growing plants—improved ventilation and watering systems.

There are usually two types of starch granules in wheat kernels—A-type and B-type. They vary in size, shape, and composition. Thus, A-type starch granules have a biconvex shape; their size is more than 10 μm and they contain more amylose than B-type granules. The second type of granule is typically round in shape and size <10 μm [26,27,28,29]. Granules are widely present in the starchy endosperm of the kernel, where they are located within a protein matrix. In our study, this was also confirmed by the granule roundness indices. We have found that the length of starch granules in kernels from the ISS was significantly longer than that from accessions in the terrestrial treatment. Perhaps, this can be partially explained by the reaction of plants to small doses of stress, as was shown in the work with the influence of low salt concentrations on kernel parameters [30].

The baking properties of wheat are closely related to the composition (two types of glucose polymer, amylose, and amylopectin), structure, and morphology of starch granules, as well as storage proteins (gliadins and glutenins) of the grain, which determine the viscoelastic properties of the dough [31,32]. In mature endosperm, protein content is higher at the periphery of the kernel and decreases towards the central part (kernel cavity), and starch granules accumulate in exactly the opposite direction [33]. In a number of studies, the kernel was divided into nine successive layers (pearling method) and confirmed that differences in the spatial distribution of chemical components affect the quality of flour. Thus, bread from layers 3–6 had a higher loaf volume, specific volume, elasticity, and organoleptic characteristics [34]. Our study revealed that there are more type B granules in terrestrial experience; that is, it can be assumed that kernels from the ISS will be slightly inferior in baking quality. Also, changes in starch content under the conditions of space were reported in works on the seeds of *Brassica rapa* [8,35], pepper plants [36], and on the roots of the watercress *Lepidium sativum* L. [37], Arabidopsis [20].

The cells of the aleurone layer are rich in protein and triacylglycerols [32]. In our work, in the ripened wheat grains on the ISS, the width of the aleurone layer cells was significantly smaller in matured wheat kernels from the ISS (Hypothesis D was confirmed) than in the terrestrial experiment; therefore, they like have less of the protein component. Reduced protein levels in *Brassica rapa* seeds obtained under space station conditions were previously reported [8,35]. Also, *Arabidopsis thaliana* seeds had 54.73% fewer protein bodies than the ground control [20]. Researchers believe this is due to the harsh conditions of microgravity, such as increased ethylene content. The lack of natural ventilation in growth chambers can easily lead plants to oxygen starvation [38] and, as a consequence, to seed hypoxia. Some symptoms of oxygen deficiency are a reduction of protein bodies, abnormal deposition of starch granules, and thickening of cell walls [39].

When analyzing the qualitative differences between kernels obtained on Earth and in the ISS, both symmetrical and asymmetrical kernels were used with an emphasis on the starch inclusions of the endosperm, which are an indicator of the quality of starch and flour for wheat. No significant differences were found in the size and arrangement of starch grains in fully developed symmetrical kernels grown both on Earth and in space flight conditions. However, differences were observed both in the connecting elements of cytoplasmic residues and in the size, location, and ratio of A and B starch inclusions in kernels with manifested asymmetry. It can be argued that if optimal conditions are created under which developmental disorders will be absent, the quality of grain and flour will not differ from those on Earth. It is possible that the cause of this phenomenon may be the peculiarities of the manifestation of the physical properties of water in weightlessness, which do not allow us to completely simulate this phenomenon. Thus, the availability of water for plants and the technical device for its provision can be key indicators for yield obtaining under space flight conditions. This is confirmed by the changes in the epidermis and hairs on the surface that we described earlier [19].

In the future, prospects are seen in accelerating the determination of the asymmetry of kernels by introducing computer scanning and developing a system for placing grains in a vertical position. We also consider it promising to study the relationship between the chemical composition of the seeds and the asymmetry of the fruits using hyperspectral and computer scanning methods. The small differences in kernel characteristics observed in two treatments to which wheat plants are poorly adapted, constant temperature, 24 h light, and low gravity, suggests that wheat cultivars better adapted to non-earth conditions would be relatively easy to breed. Such plants would be better adapted to space conditions and would not require some genes involved in resistance to abiotic and biotic stresses. Because some of the genes used to limit crop losses on the earth do have adverse effects on plant vigor.

## 4. Materials and Methods

### 4.1. Plant Material

For the study, spring wheat of the Super Dwarf cultivar (*Triticum aestivum* L., 2n = 42) was used. This variety (CMH79.481–1Y8B-2Y-2B-OY) was selected by Dr. Bruce G. Bugbee in 1984 at the International Maize and Wheat Improvement Center (CIMMYT) in Mexico, because the variety showed high adaptive potential to hydroponics, high CO_2_ content, and artificial lighting [11]. Super Dwarf plant heights are 15–20 cm in the field and 25–30 cm in growth chambers, making it attractive for growing in space bioregenerative life support systems. The heredity of Super Dwarf contains the germplasm of *T. sphaerococcum*, so the kernels of this cultivar are approximately two times shorter and thicker than the kernels of the usual varieties [11].

### 4.2. Conditions for Obtaining a Harvest from Super Dwarf Wheat

**Figure 11:**
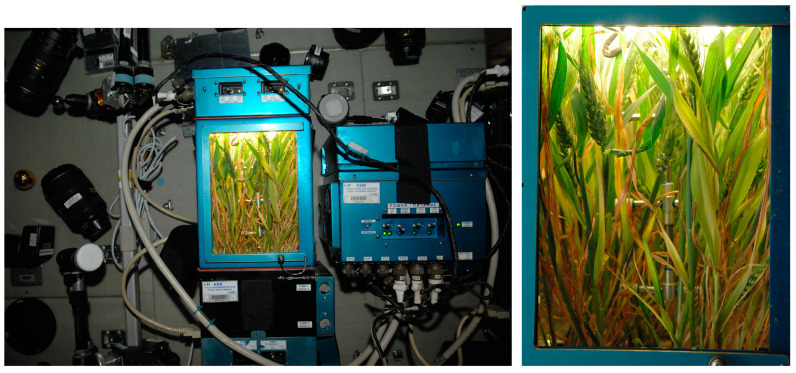
Appearance of wheat plants in the Lada greenhouse on the ISS 10 June 2011.

On 8 August 2011, cosmonaut S.A. Volkov (ISS-28/29 expedition) sowed the Super Dwarf grains in two Lada greenhouses on the Russian segment of the International Space Station (ISS) (Figure 11).

The growth chamber consists of a module for roots with Turface substrate (AIMCOR, Buffalo Grove, IL, USA) and a leaf chamber for the above-ground parts of plants with mirror walls and fluorescent lamps [13]. Watering was carried out when the substrate humidity dropped below 90–96%; Osmokote long-acting fertilizers were applied once in an amount of 15 g for the entire volume of the substrate in the chamber. The temperature in the greenhouse was 22 °C, the photoperiod was around the clock (Figure 11). The ISS-grown wheat plants, although they showed some deviation from vertical growth, looked quite normal, retaining their elongation from roots to ears. Upright growth of the wheat plant on the ISS is likely caused by phototropism [40].

**Figure 12:**
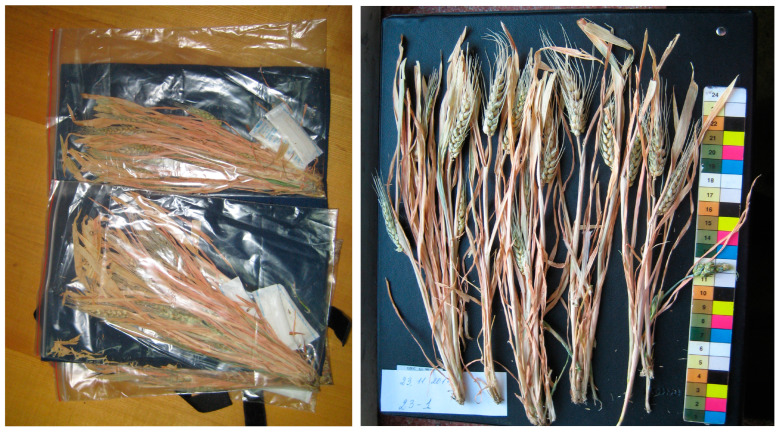
Harvested wheat plants delivered from the ISS on 23 November 2011.

On 17 November 2011, the mature plants were harvested and placed into two plastic bags with silica gel fillers to remove residual moisture and delivered to Earth on the Soyuz TMA-02M manned transport spacecraft (Figure 12).

Wheat kernels of the same cultivar Super Dwarf, obtained in a similar Lada chamber in the ground laboratory, served as a control. To check the safety of kernels grown in 2011 using a hyperspectral camera, wheat was grown on the ground in 2023 under similar conditions.

One hundred kernels from the ISS (in two bags of 50 pieces each) and 85 kernels of the control variant in paper bags were obtained from the Institute of Medical and Biological Problems (Moscow, Russia) in 2022.

### 4.3. Determination of the Asymmetry of Kernels in a Non-Traumatic Manner

#### 4.3.1. Method of Perpendiculars to the Axis of Symmetry at a Distance of 25, 50, and 75% of the Kernel Length from the Base

**Figure 13:**
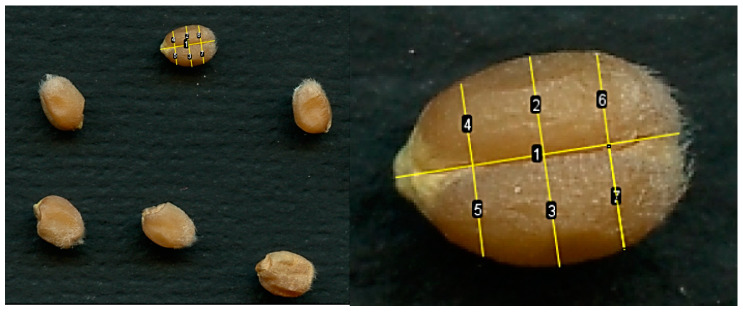
Location variants of kernels that fell onto the scanning surface by the crease zone. Line 1—axis of kernel symmetry; Lines 2 and 3—the length of the left and right sides of a kernel, intersecting the symmetry axis at the point of 50% of the length of the kernel; lines 4 and 5—the length of the left and right sides of the kernel, intersecting the axis of symmetry at the point of 25% of the length of the kernel; lines 6 and 7—the length of the left and right sides of the kernel, intersecting the axis of symmetry at the point of 75% of the kernel length from the base.

The kernels of each option were individually poured from paper bags onto the scanning surface of an Epson Perfection V550 Photo scanner, and the kernels were covered with black thick paper on top. The distance between the kernels was increased or decreased so that they did not touch, and their shadows did not creep onto each other when processed in ImageJ v2023. Wherein, the kernels were not turned over. Images were acquired at 600 dpi resolution. The kernel may fall onto the scanning surface sideways or onto the area where the two sides of the endosperm meet (crease), taking into account the uneven balance of weight inside the kernel. If it falls to one side, it will not be possible to establish asymmetry, since the symmetry line of the crease will not be reflected correctly (Figure 13). When placing the kernel by the crease to the scanning surface, the length of the left and right sides of the kernel was measured in the middle (crossing approximately 50% of the length of the symmetry axis of the kernel), and closer to the edges (approximately at the level of 25 and 75% of the length of the symmetry axis of the kernel from the kernel base).

First, the measurements of 15 kernels were recorded, and then the sample was increased to 30 kernels (15 control kernels, 15 from ISS). The presence of significant differences between the right and left sides of the kernel was checked using the Kolmogorov–Smirnov method and additionally using the Mann–Whitney test.

#### 4.3.2. Method of Perpendiculars to the Axis of Symmetry through the Point of Attachment (Base) of the Kernel and the Zone of Crease

**Figure 14:**
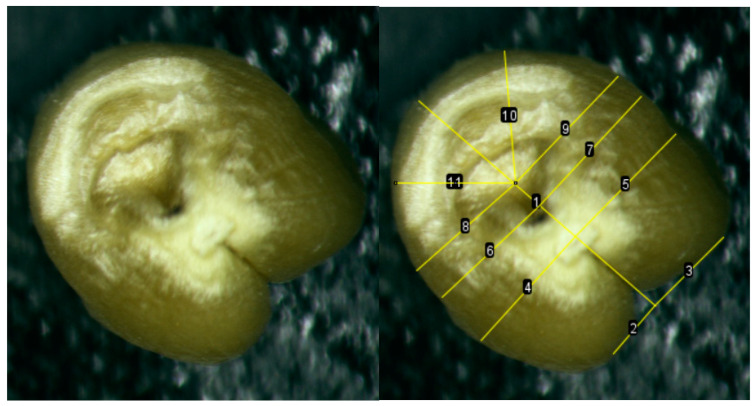
Perpendiculars to the axis of symmetry at the base of the kernels (line 1). Where lines 2 and 3 (or for schematic purposes L1 and L1’) are the distance from the vertices of the lobes to the axis of symmetry; lines 4 and 5 (or L2 and L2’)—the distance from the edges to the axis through the attachment point; lines 6 and 7 (or L3 and L3’)—from the edges to the axis through the hole; lines 8 and 9 (or L4 and L4’)—from the edges to the axis through the radicle; lines 10 and 11 (or L5 and L5’)—the distance from the edge to the axis at an angle of 45%.

The kernels were placed vertically, gluing the kernels to the sticky tape with the brush area. Each kernel was assigned its own serial number, which remained behind the kernel at subsequent stages of the study. The image from the microscope was displayed on a computer screen and photographs were taken using the RisingView program. Next, measurements were performed in the ImageJ program. If the kernel has a fairly clearly defined attachment point, an opening between the attachment point and the radicle, the radicle, then the distance from the vertices of the lobes to the axis of symmetry (L1 and L1’), from the edges to the axis through the attachment point (L2 and L2’), from the edges to the axis through the hole (L3 and L3’), from the edges to the axis through the radicle (L4 and L4’), the distance from the edge to the axis at an angle of 45% (L5 and L5’), because visually there is often asymmetry here (Figure 14).

**Figure 15:**
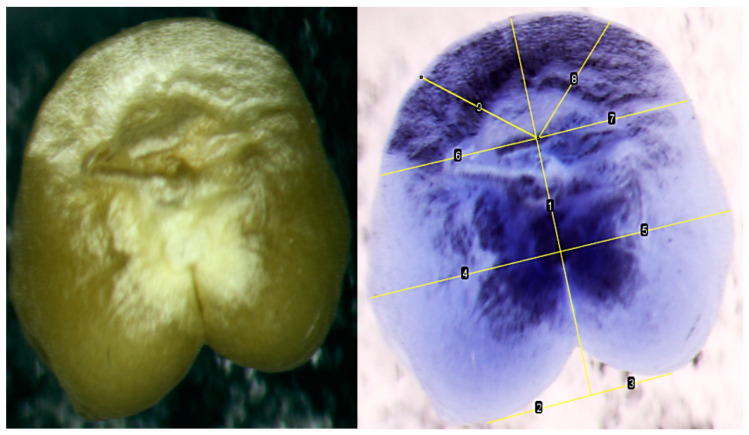
Perpendiculars to the axis of symmetry at the base of the kernels (line 1). Where lines 2 and 3 (or schematically L1 and L1’ for schematic purposes) are the distance from the vertices of the lobes to the axis of symmetry; lines 4 and 5 (or L2 and L2’)—the distance from the edges to the axis through the attachment point; lines 6 and 7 (or L4 and L4’)—from the edges to the axis through the radicle; lines 8 and 9 (or L5 and L5’)—the distance from the edge to the axis at an angle of 45%.

If the hole was not expressed, then all distances were measured except for the lines that were perpendicular to the axis of symmetry through the hole, i.e., without L3 and L3’. If the protruding radicle was difficult to distinguish in the photo, then we used the Invert function in ImageJ to obtain a blue-colored photo in which the relief was more clearly visible (Figure 15).

In this way, the lengths of the left and right sides of the base of the kernel were measured for 100 accessions from the ISS and 85 control accessions. The significance of the differences was determined using the *t*-test method.

### 4.4. Determining Descriptive Statistics for the Sample

Each kernel was placed on the adhesive tape with its back facing up, i.e., the junction of the two sides of the endosperm downwards according to the previously assigned serial number. Next, we obtained images from an Epson Perfection V550 Photo scanner (Epson, Suwa, Japan) with a resolution of 600 dpi. In the ImageJ program (National Institutes of Health, Bethesda, MD, USA), we processed photographs and measured the area of the kernel (mm^2^), perimeter (mm), roundness index, length (mm), width (mm), depth of the kernel (from the point of the junction of the two lobes of the kernel to the abdominal side at cross section, mm), depth of crease (the distance from the top of the junction of the two sides of the kernel to the point of attachment of the kernel). The weight of the kernels was determined with an accuracy of 0.0001 g. The distribution of a sample was checked for normality using the Kolmogorov–Smirnov method. The significance of the differences between the control sample and kernels from ISS was determined using the *t*-test method in SPSS Statistics 25.

### 4.5. Calculation of Asymmetry Indicators

The direction of asymmetry was calculated as the ratio of the length of one of the sides to the sum of the lengths of the left and right sides [30]. For example, the direction of asymmetry from the vertices of the lobes to the axis of symmetry (Figure 3 and Figure 4) was found as L1/(L1 + L1’). All calculated values of indices of directions were in the range from 0 to 1. An index of 0.5 indicates symmetry of the sides, and so, with an assumption of 5%, it can be assumed that indices of 0.48, 0.49, 0.51, and 0.52 also indicate symmetry. Calculated values from 0 to 0.48 show a shift of symmetry to the left, and values from 0.53 to 1 are considered to be right-directed. If 3–5 indices out of 5 have the same direction, then we take this direction as directionality, for example, 3 indices are left-directed, 1 index is right-directed, and 1 more indicates symmetry, then we consider the kernel to be left-directed. The direction of asymmetry to the left was found to be characteristic of kernels that were located on the right in a simple ear [18].

Indices of relative asymmetry and fluctuating asymmetry of kernel were calculated using the previously applied method [18]. The relationship of asymmetry indices with the main parameters of kernel was assessed using the Spearman rank correlation method in SPSS Statistics 25.

### 4.6. Comparison of Asymmetry Indices Obtained Using Both a Kernel Section and a Non-Traumatic Method

Early studies showed that the sufficient sample size to establish the asymmetry of kernel was 15 kernels per variant [18]. In present work, 20 kernels per variant were used (ISS and control), including two groups—kernels with a hole between the point of attachment of the kernel and the radicle and without such a hole. The selection of kernels was carried out randomly due to the function in SPSS “formation of a random sample of a given size”. Samples with serial numbers selected by the program were cut in the middle across the kernel. Relative asymmetry indices, asymmetry directional indices, and the value of fluctuating asymmetry were determined [18]. Comparison of indices from the kernel section and using a non-traumatic technique was carried out using the Kruskal–Wallis and Mann–Whitney method in SPSS.

### 4.7. Scanning Electron Microscopy

Microscopy of the kernels was performed using a Camscan scanning electron microscope (Cambridge Instruments, Cambridge, UK). The kernels were placed on a metal stage, treated with a thin layer of gold and palladium, and then photos of the endosperm were taken using a scanning electron microscope (10 nm, ×1000). Using the resulting photographs, the length and width of starch granules on sections of kernels and on cut prints (starch granules are separate from each other) were measured. Using the resulting photographs, the length and width of starch granules on sections of kernels and on prints of sections (starch granules are separate from each other) were measured. Forty kernels were studied, of which 10 kernels were filled kernels from the ISS, 10 kernels were puny kernels from the ISS, and 20 kernels were from Earth experiment. On each kernel, five photographs were randomly taken with magnification of 1000 and 1500 times. The normality of the distribution was checked by the Kolmogorov–Smirnov method, and the means were compared using the Kruskal–Wallis analysis of variance.

### 4.8. Hyperspectral Imaging

Hyperspectral images were obtained using the Synergotron Fenoscaner (Synergotron, Zagreb, Croatia) research complex, which includes a hyperspectral camera with scanning technology equipped with a branching module with a transmission grating with high diffraction efficiency and a camera based on a high-sensitivity matrix. This, coupled with the built-in scanning technology and auxiliary camera, overcomes the complex problems encountered with conventional hyperspectral cameras, such as external longitudinal scanning mechanism and difficult focus.

Spectral range: 400–1000 nm, spectral resolution above 2.5 nm, 300 spectral channels. The image resolution was 1920 × 1920. Every 10 measurements, calibration measurements were taken on a white calibration panel coated with BaSO_4_.

Image analysis was carried out using Gelion software (Gelion 1.0b.sh., Moscow, Russia—gelion.agro.msu.ru). Analysis of the approximation of the spectral curve of kernels to the reference values of the spectral profiles of the coloring substances of the kernel, specifically in this case anthocyanins (AnC) were analyzed, identified by the type of the spectral curve—the dependence of the spectral brightness on the wavelength. At the same time, the reference spectra were brought to a single brightness scale—reflection coefficients. Reflectance coefficient (r) is the ratio of reflected radiation to incident radiation in the direction perpendicular to the surface. The reflectance in a certain direction relative to the Earth’s surface is called spectral albedo.

The NDVI index (normalized relative vegetation index)—a simple indicator of the amount of photosynthetically active biomass—was calculated using the formula (800 nm − 680 nm)/(800 nm + 680 nm). Vegetation index values range from 0.20 to 0.95.

## 5. Conclusions

The work presents, for the first time, data on the asymmetry of wheat kernels obtained in near space conditions, and also indicates the relationship of this parameter with other important characteristics of the kernel. It has been found that there were no significant differences between the main parameters of the kernels from the wheat plants grown on the ISS and on Earth. The number of type B starch granules in kernels from ISS was 5–8% less than in those obtained on Earth, and the width of the cells of the aleurone layer in kernels from ISS was significantly inferior to that obtained on Earth. Despite the fact that many parameters of the grains were statistically the same, a decrease in the protein component in the endosperm indicates a likely increase in stress components, gravity loss, water stress, and possibly some tissue hypoxia during the formation of space generation seeds; therefore, the growth chambers should be modified for better aeration.

It should be noted that growing plants from seeds in space opens up prospects for sustainable food production and the stability of artificially created ecosystems not only during long space flights, but also in other closed-cycle conditions; for example, when creating stationary systems with extreme conditions on other planets, at base stations in the Arctic and Antarctic, high mountain areas, or at underground or underwater objects. The data presented make it possible to obtain valuable information about the asymmetry of the shape, protein, and starch composition of the kernel in the space environment. Continued research in this area will contribute to the development of space agriculture and the creation of a self-sustaining space habitat. The phototropic responses of plants are likely the main factor preventing drastic gravitational responses in space.

## Figures and Tables

**Table 1:** Descriptive statistics of the main parameters of kernels (*p* = 0.05) and testing the significance of differences using the *t*-test method.

Parameters	Experimental Conditions	Arithmetic Mean ± Standard Deviation	Significant Differences between Samples (*t*-Test)	The Coefficient of Variation, %
Weight of kernel, g	Lada ISS	0.0362 ± 0.0044	Confirmed	12
	Lada Earth	0.0376 ± 0.0046		12
Kernel area, mm^2^	Lada ISS	11.64 ± 1.22	Confirmed	11
	Lada Earth	13.30 ± 1.42		11
Kernel perimeter, mm	Lada ISS	19.92 ± 3.17	No differences identified	16
	Lada Earth	20.49 ± 2.20		11
Kernel length, mm	Lada ISS	4.42 ± 0.38	Confirmed	9
	Lada Earth	4.87 ± 0.38		8
Kernel width, mm	Lada ISS	3.36 ± 0.19	Confirmed	6
	Lada Earth	3.47 ± 0.20		6
Kernel roundness	Lada ISS	0.39 ± 0.10	No differences identified	26
	Control	0.40 ± 0.09		23
Kernel depth, mm	Lada ISS	3.38 ± 0.22	No differences identified	7
	Lada Earth	3.42 ± 0.24		7
Recess kernel depth, mm	Lada ISS	0.88 ± 0.23	No differences identified	26
	Lada Earth	0.93 ± 0.31		34

The kernels from the two treatments are relatively homogeneous because coefficients of variation are below 15%.

**Table 2:** Variation coefficients of characteristics of starch granules, %.

	Type A Starch Granules				Type B Starch Granules			
	Space Station (Lada ISS)		Control (Lada Earth)		Space Station (Lada ISS)		Control (Lada Earth)	
	Filled	Puny	Filled	Puny	Filled	Puny	Filled	Puny
starch granule length	49	41	52	49	29	19	22	20
starch granule weight	54	46	49	47	40	21	33	24
granule roundness index	11	12	14	11	22	11	20	11

**Table 3:** Average values of the length and width of starch granules in kernels of wheat plants grown on the ISS and in the ground experiment.

Experimental Conditions	Quality Wheat Kernel	Length of Starch Granule Type A, μm	Length of Starch Granule Type B, μm	Width of Starch Granule Type A, μm	Width of Starch Granule Type A, μm	Roundness Index of Starch Granule Type A	Roundness Index of Starch Granule Type B
ISS	Filled	4.32 ± 2.13 ^b^	17.15 ± 5.03 ^b^	3.91 ± 2.12 ^a^	14.28 ± 5.76 ^b^	0.88 ± 0.09 ^a^	0.81 ± 0.18 ^a^
	Puny	4.10 ± 1.69 ^b^	18.45 ± 3.48 ^b^	3.66 ± 1.70 ^a^	14.23 ± 2.99 ^b^	0.85 ± 0.11 ^a^	0.77 ± 0.09 ^a^
Earth	Filled	3.37 ± 2.05 ^a^	17.77 ± 3.98 ^b^	3.38 ± 1.65 ^a^	14.43 ± 4.71 ^b^	0.82 ± 0.12 ^a^	0.79 ± 0.16 ^a^
	Puny	3.44 ± 1.68 ^a^	15.32 ± 3.06 ^a^	3.18 ± 1.50 ^a^	12.63 ± 3.02 ^a^	0.86 ± 0.10 ^a^	0.82 ± 0.09 ^a^

Letters indicate groups without significant differences based on comparison of means using the Kruskal–Wallis analysis of variance (*p* ≤ 0.05).

**Table 4:** Average values of the length and width of cells of the aleurone layer on sections of kernels.

Experimental Conditions	Kernel Wheat Quality	Average Length of Cell Aleurone Layer, μm	Average Width of Cell Aleurone Layer, μm
Lada ISS	Filled	38.53 ± 10.72 ^a^	29.33 ± 5.85 ^a^
	Puny	35.04 ± 11.07 ^a^	39.38 ± 11.18 ^b^
Lada Earth	Filled	37.51 ± 7.44 ^a^	42.29 ± 8.90 ^b^
	Puny	43.41 ± 10.07 ^a^	43.11 ± 11.09 ^b^

Letters indicate groups that do not have significant differences between themselves (*p* ≤ 0.05, ANOVA with post hoc Scheffe test).

## Data Availability

Data are contained within the article.
